# MRI-based virtual radiographs of the pelvis for assessment of acetabular morphology

**DOI:** 10.1186/s41747-026-00835-7

**Published:** 2026-11-06

**Authors:** Jose Andres Roshardt, Julien Hirt, Malin Kristin Meier, Till Dominic Lerch, Alexander Frank Heimann, Moritz Tannast, Armando Hoch, Stefan Sommer, Reto Sutter, Simon Damian Steppacher, Florian Schmaranzer

**Affiliations:** 1 Department of Orthopaedic Surgery and Traumatology, Inselspital, University of Bern, Bern, Switzerland; 2 Graduate School for Cellular and Biomedical Sciences, University of Bern, Bern, Switzerland; 3 Department of Orthopaedic Surgery and Traumatology, HFR Cantonal Hospital, University of Fribourg, Fribourg, Switzerland; 4 Department of Diagnostic, Interventional and Paediatric Radiology, Inselspital, University of Bern, Bern, Switzerland; 5 Department of Orthopaedics, Balgrist University Hospital, University of Zurich, Zürich, Switzerland; 6 Swiss Center for Musculoskeletal Imaging (SCMI), Balgrist Campus, Zurich, Switzerland; 7 Swiss Innovation Hub, Siemens Healthineers International AG, Zurich, Switzerland; 8 Department of Radiology, Balgrist University Hospital, University of Zurich, Zürich, Switzerland

**Keywords:** Deep learning, Developmental dysplasia of the hip, Femoroacetabular impingement, Hip magnetic resonance imaging, Hip radiography

## Abstract

**Objectives:**

We generated magnetic resonance imaging (MRI)-based virtual radiographs (VRs) from hips with/without intra-articular contrast and compared them with conventional anteroposterior pelvic radiographs (CR) regarding measurement bias, interobserver and intraobserver agreement, and diagnosis of acetabular deformities; we also assessed hip deformity prevalence on VR in asymptomatic participants.

**Materials and methods:**

Retrospective study of 50 patients (100 hips; mean age 34 years) with femoroacetabular impingement or hip dysplasia undergoing CR and unilateral magnetic resonance arthrography. Additionally, 53 asymptomatic participants underwent noncontrast MRI. Segmentation of the osseous pelvis was performed on T1-weighted volume interpolated breathhold examination (VIBE) Dixon MRI using 3D nnU-Net, pseudo-computed tomography generation, and cone-beam projection to generate VR. Acetabular coverage (lateral center edge angle (LCE), acetabular index (AI), anterior/posterior coverage, extrusion index (EI)), and acetabular version were compared between CR and VR. Interobserver agreement was assessed using intraclass correlation coefficients (ICC) and *κ* statistics; bias with Bland–Altman analysis.

**Results:**

Interobserver agreement for VR ranged from moderate to almost perfect (ICC/*κ* 0.60–0.94), except for the AI. LCE showed no significant bias with ( − 0.2° ± 2.5, *p* = 0.663) or without contrast ( − 0.2° ± 2.2, *p* = 0.530). Systematic differences occurred for AI, EI, anterior/posterior coverage, and ischial spine sign; 57–75% of discrepancies were within CR interobserver variation. Diagnostic classification showed substantial agreement between CR and VR (*κ* = 0.62), comparable to CR interobserver agreement (*κ* = 0.70). In asymptomatic participants, 13% had acetabular dysplasia, 68% normal coverage, and 19% overcoverage.

**Conclusion:**

MRI-based VR provides acetabular morphology assessment comparable to CR and enables radiation-free evaluation in symptomatic patients and asymptomatic populations.

**Key Points:**

***Question*** Can MRI-based virtual radiographs reproduce conventional radiographic measurements of acetabular morphology, enabling radiation-free two-dimensional assessment in symptomatic patients and asymptomatic populations?

***Findings*** The lateral center-edge angle showed no significant bias between virtual and conventional radiographs; diagnostic classification of acetabular deformities agreed substantially (κ = 0.62), comparable to conventional radiograph interobserver agreement.

***Relevance Statement*** MRI-derived virtual radiographs can provide acetabular measurements comparable to conventional radiographs without ionizing radiation, supporting integration into three-dimensional surgical planning and enabling assessment in young patients and population-based cohorts where repeated radiographs are undesirable.

**Graphical Abstract:**

**Figure:**
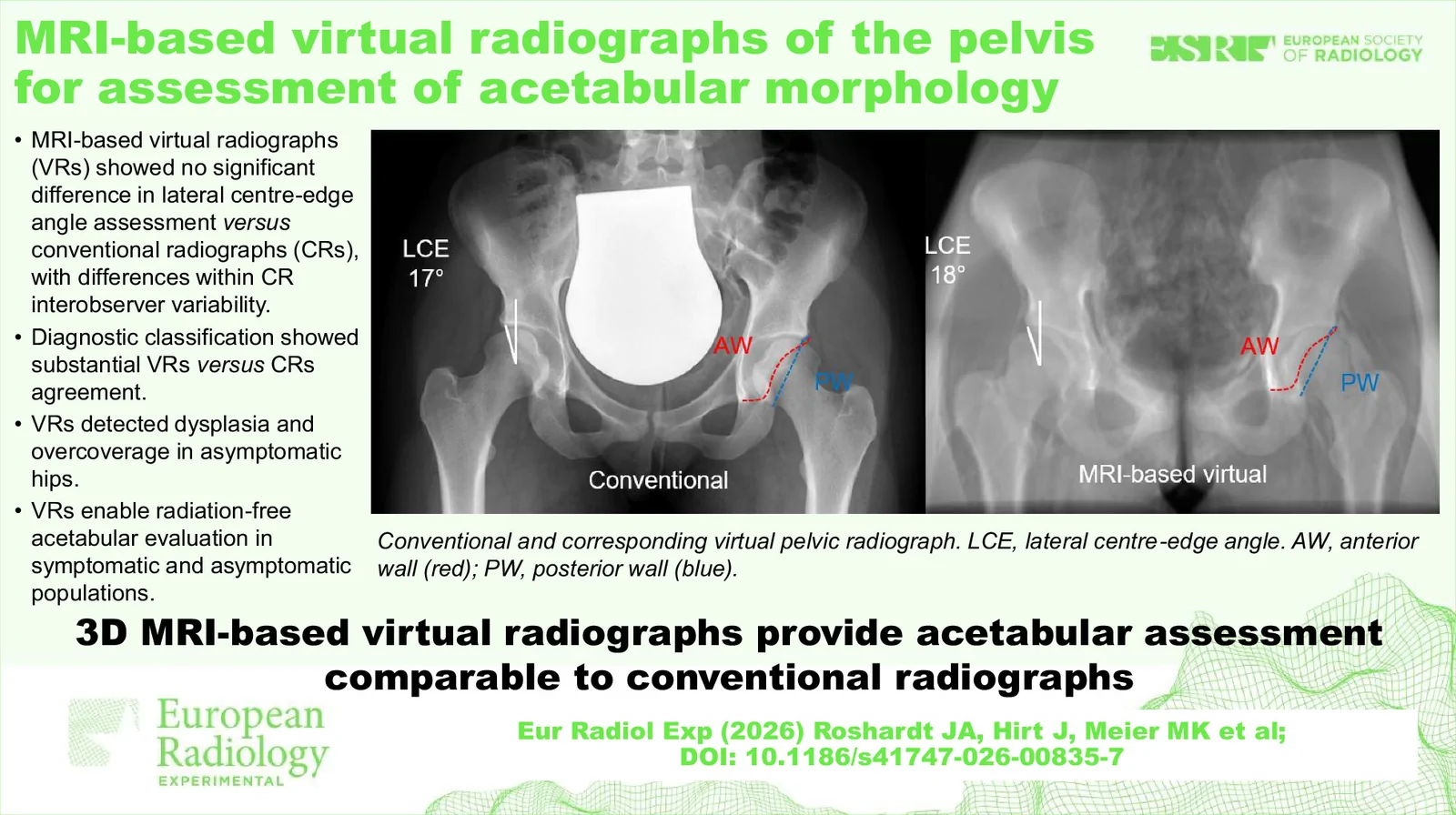

## Introduction

Femoroacetabular impingement (FAI) and developmental dysplasia of the hip (DDH) are major causes of hip pain and early-onset osteoarthritis, particularly in young, active patients [1, 2]. Diagnostic evaluation traditionally relies on clinical assessment combined with imaging [3]. Conventional radiographs (CRs) remain the standard for assessing acetabular coverage and version, as established criteria for diagnosing acetabular deformities are radiograph-based [4].

Computed tomography (CT) is commonly used for 3D visualization but involves ionizing radiation, which is problematic in young patients and research settings [1, 5]. Magnetic resonance imaging (MRI) eliminates radiation exposure and provides superior soft tissue contrast. Recent advances in artificial intelligence enable fully automated 3D segmentation of the osseous pelvis from MRI [6, 7]. These 3D models can be used for anatomical assessment and dynamic virtual impingement simulation to support surgical planning [6, 7]. To integrate this 3D workflow with established radiographic criteria, the generation of virtual radiographs (VRs) from segmented MRI models is required. This approach would allow surgeons to simulate the surgical corrections on standard angular measurements preoperatively, ensuring that the 3D correction translates to the desired two-dimensional (2D) radiographic outcome. Similar to CT-based VR, segmented 3D MRI may allow the generation of VR, eliminating patients' exposure to ionizing radiation while seamlessly integrating into our existing 3D planning pipeline [3, 8]. Furthermore, such a workflow enables radiation-free use in population-based studies of hip deformities and degeneration. VRs derived from CT have previously been generated and validated against CR [2]. In contrast, MRI-based VR of the pelvis has not yet been validated against CR, which is the rationale for the present study. Because intra-articular contrast may alter the depiction of the osseous boundaries used for segmentation, we additionally compared VR generated from hips with and without intra-articular contrast.

This study aims to evaluate the feasibility of generating MRI-based VR generated from hips with and without intra-articular contrast and compare them to CR in terms of measurement bias, inter- and intraobserver agreement, and variation in radiographic diagnosis of acetabular deformities. Furthermore, we aim to evaluate the prevalence of hip deformities based on VR in a cohort of young asymptomatic participants.

## Methods

The study was approved by the local ethics committee (Kantonale Ethikkommission Bern, Switzerland; KEK 2024-01833/2022-01508) and performed in accordance with institutional and international guidelines. All patients provided general consent for the secondary use of their clinical data. Asymptomatic participants provided written informed consent for the use of their imaging data. First, we conducted a retrospective feasibility study for generating MRI-based VR of the pelvis, using a validated deep learning method for bone segmentation [3] combined with image post-processing algorithms for the generation of a pseudo CT and cone-beam projection [4]. Second, we included prospectively collected asymptomatic participants to define the prevalence of hip deformities based on the newly developed workflow for VR.

### Participant selection

**Fig. 1:**
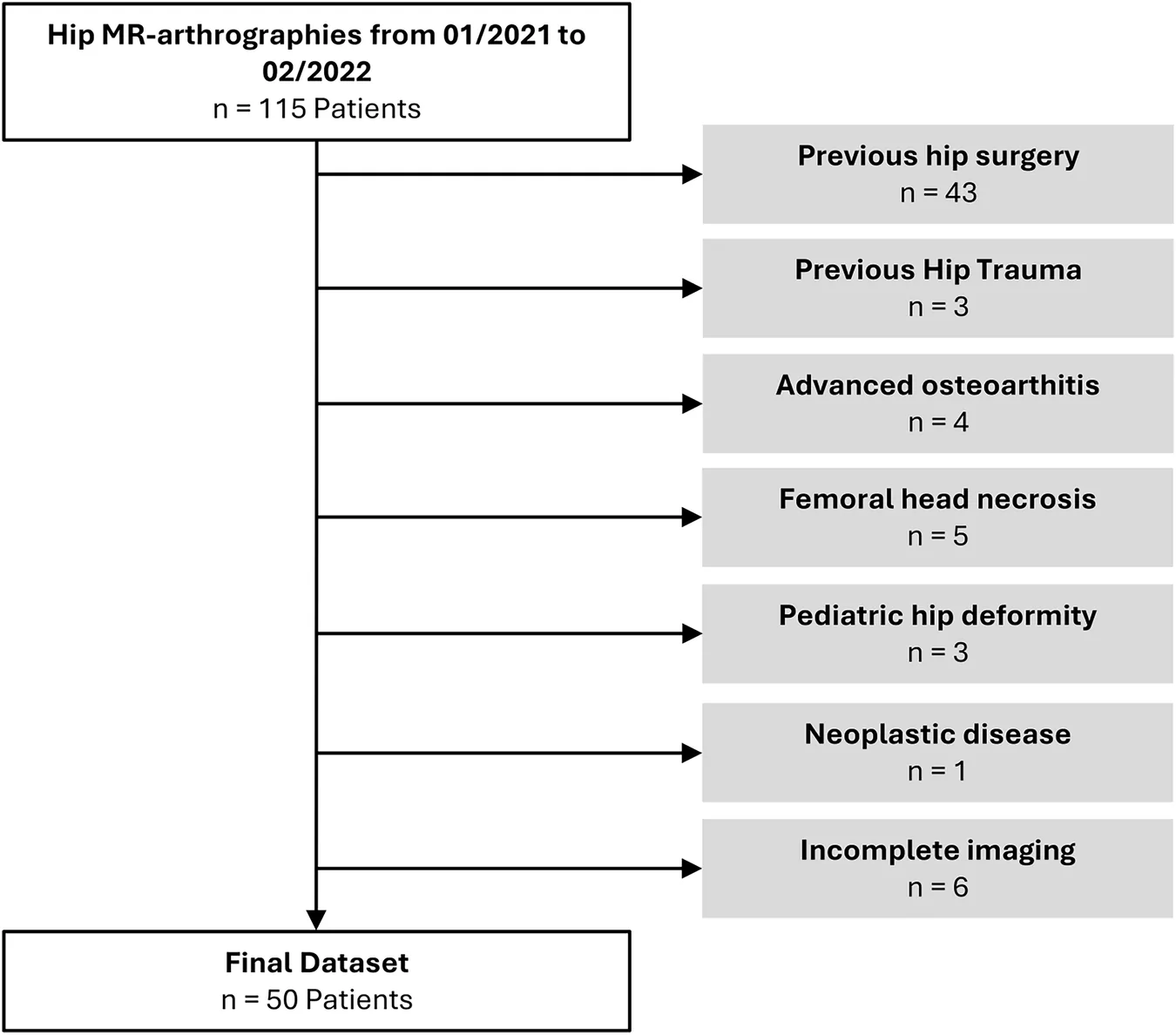
Flowchart of patient inclusion and exclusion

Patients with hip pain were referred to the outpatient clinic of a tertiary orthopedic center (Inselspital, University Hospital Bern) for diagnostic imaging according to the institutional routine protocol between January 2021 and February 2022. Inclusion criteria were symptomatic FAI or DDH, age ≥ 18 years, and imaging workup including CR and MR arthrography with a 3D T1-weighted volume interpolated breathhold examination (VIBE) Dixon sequence. Patients were excluded in case of previous hip surgery, incomplete imaging protocol, pediatric hip deformities (history of slipped capital femoral epiphysis/Perthes disease), posttraumatic hip deformities, femoral head necrosis, neoplastic conditions, advanced osteoarthritis (Tönnis grade > 1) (Fig. 1). For the asymptomatic participants inclusion criteria were no history of hip pain and absence of hip pain on a clinical examination performed by an orthopedic resident (J.R.). Asymptomatic volunteers with no current or prior history of hip pain were recruited prospectively. The exclusion criteria were identical to those used for the symptomatic cohort.

### Image acquisition

Conventional radiographs of the pelvis were acquired with patients in the supine position and 15° internal leg rotation, which is the standard protocol at our institution. The film-focus distance was 1.2 meters, and the central beam was directed at the midpoint between the anterosuperior iliac spines and the superior border of the symphysis [5]. Arthrography was performed under fluoroscopic guidance. MRI was performed using a standardized protocol at 3 T (MAGNETOM Vida, Siemens Healthineers) with patients supine and toes taped together in 10 to 15° internal rotation. Large flexible surface coils were used, and multiplanar proton density-weighted sequences were acquired for assessment of joint damage. 3D T1-weighted axial VIBE Dixon sequences (in-phase and water-only image reconstructions) covering the pelvis and distal femoral condyles to measure femoral torsion [6]. For this study, the VIBE Dixon sequence covering the pelvis was used for automatic bone segmentation, as it provides 3D images with 1 mm slice thickness and offers different image contrasts with a rapid acquisition time (41 s). [7].: Imaging parameters included a repetition time 3.94 ms, echo time 1/2 1.27 ms/2.5 ms, flip angle 9°, slice thickness 1.0 mm, field of view 312 × 400 mm^2^, matrix 175 × 320, voxel size 1.2 × 1.2 × 1.0 mm^3^. In the asymptomatic participants, no radiographic imaging was performed due to ethical reasons, and non-contrast MRI of the hip was performed at 3 T (MAGNETOM Prisma, Siemens Healthineers) using the same protocol.

### Virtual radiographs of the pelvis

Generation of VR consisted of three steps: deep learning-based segmentation of the osseous pelvis, generation of pseudo-CT images, and cone-beam projection.

**Fig. 2:**
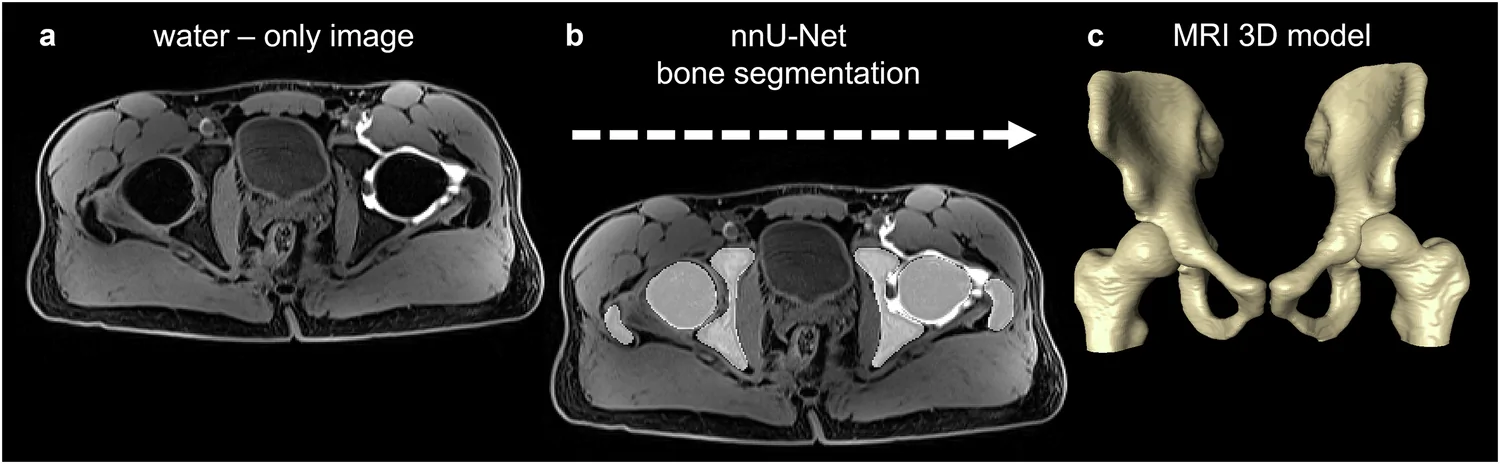
The pipeline for the automated pelvic bone segmentation is shown using the (**a**) water-only VIBE Dixon images as input. **b** A nnU-Net is then used to mask the pelvic bone and proximal femur, and (**c**) create 3D models

First, a previously validated deep-learning approach for fully automated pelvic bone segmentation achieving high segmentation accuracy (dice coefficients of 94% for pelvic bone and 97% for the proximal femur) was adapted using the self configurating nnU-net framework from Isensee et al [3, 8]. For automated segmentation, the water-only VIBE Dixon images, exhibiting a hypointense appearance of the cancellous and cortical bone, were used as input (Fig. 2). Automated bone segmentation masks and 3D models were visually checked for segmentation errors by an orthopedic resident (J.R.) with 5 years of experience in hip imaging and by a fellowship-trained musculoskeletal radiologist (F.S., 10 years of experience). No errors requiring corrective action were detected. A relevant segmentation error was defined as any deviation of the bone mask from the cortical boundary at the acetabular rim, anterior or posterior wall (P.W.), or femoral-head contour judged sufficient to alter a measurement. Masks were reviewed (independently and then by consensus), blinded to the CRs, with specific attention to the landmarks used for projection and measurement.

**Fig. 3:**
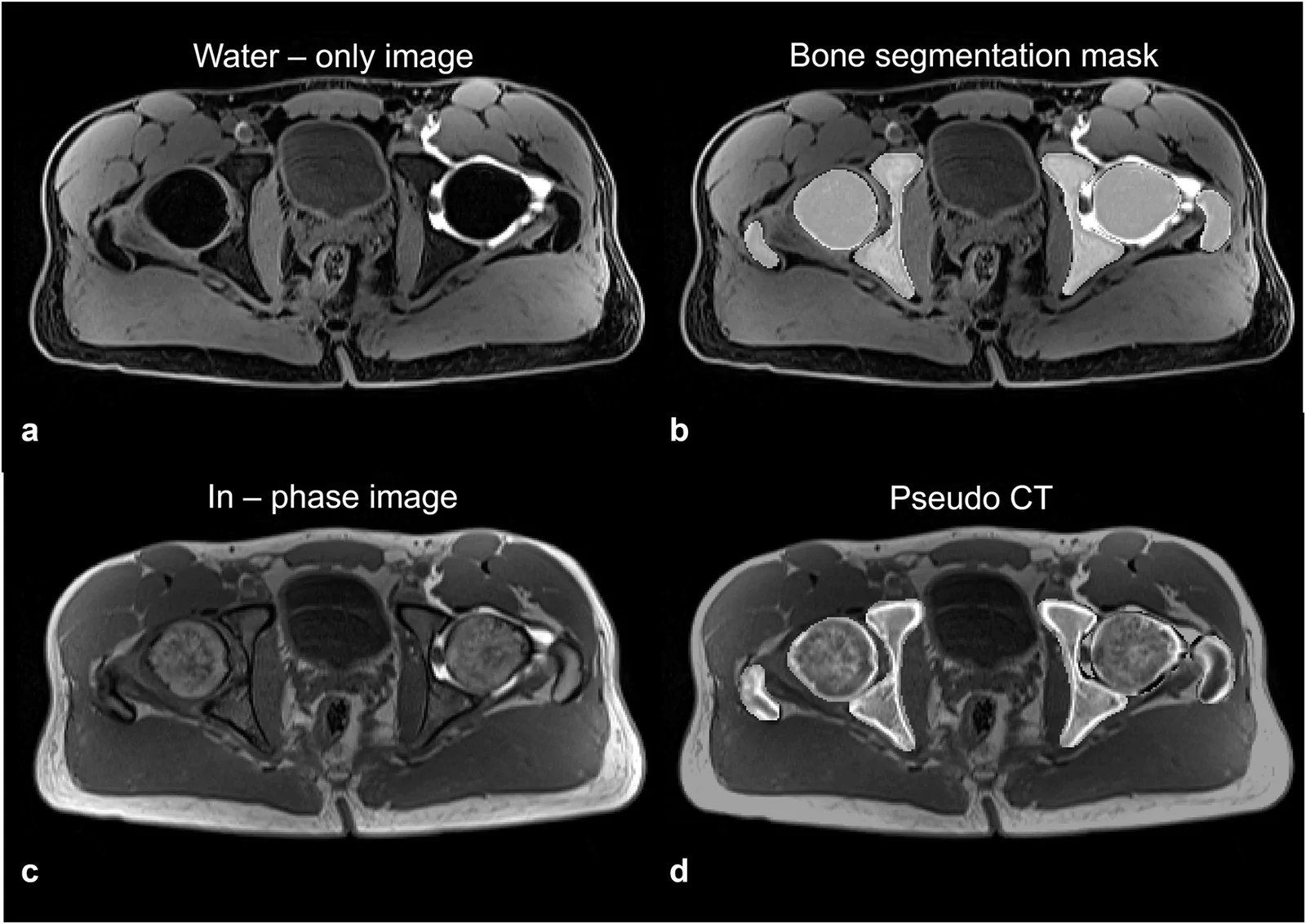
**a** Water only VIBE Dixon images are used as input for the nnU-Net, which masks out the pelvic bones (**b**). In a second step, the segmented mask of the pelvis and proximal femur is then improved using the in-phase VIBE Dixon image as input. **c** On these images, the cortical bone remains a hypointense, dark sclerotic line, whereas the cancellous bone has more signal due to the fat of the bone marrow. This difference is accentuated using a mathematical transformation followed by inversion of the bone contrast to create a pseudo-CT of the pelvis (**d**)

Second, segmentation masks of the pelvic bone and the proximal femur were applied to the in-phase images, which are inherently co-registered to the water-only images. The contrast of the in-phase images was then modified within a dilated bone segmentation to generate a pseudo-CT by using equation [1] implemented in MATLAB Version R2018b (The MathWorks, Natick, Massachusetts, USA) (Fig. 3).

1 $${pseudoCT}\left[{mask}==1\right]=2\cdot (1-\sqrt{{MR}\left[{mask}==1\right]})$$

*MR* describes the in-phase input, where *mask* is the dilated bone mask from the previous step which is used to generate the pseudo CT *(pseudoCT)* which exhibits hyperintense cortical bone of the pelvic bones and proximal femur (Fig. 3).

Third, the pseudo-CT images were used as input to a cone-beam projection algorithm implemented in MATLAB based on Kim et al [4]. This includes a virtual cone beam radiographic source positioned at a 1.2-m distance from the 3D model and is directed to the midpoint of the symphysis and a line connecting the anterosuperior iliac spines, similar to CR (Supplementary Fig. S1).

### Outcome parameters

**Fig. 4:**
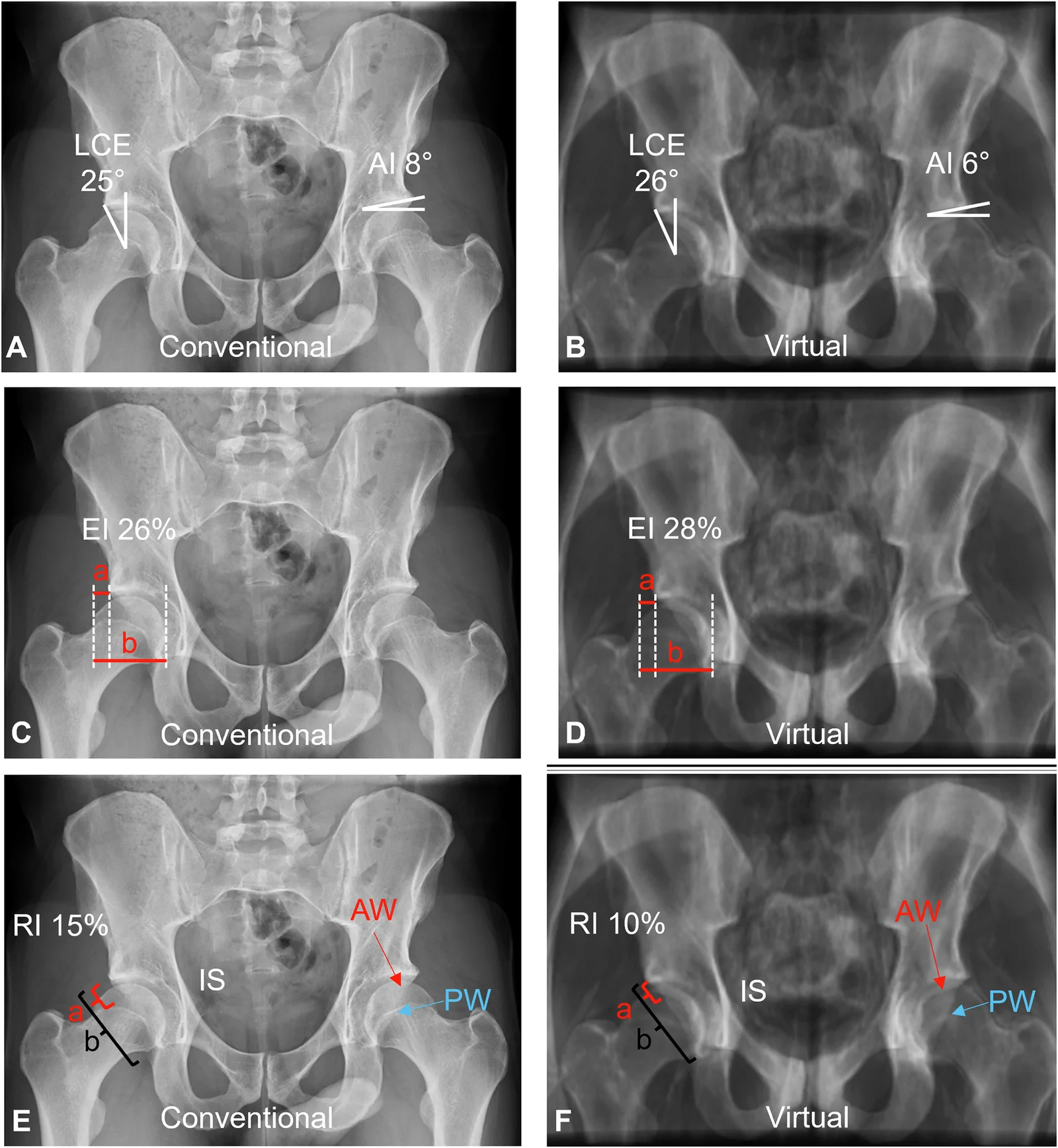
Conventional (**A**, **C**, **E**) and corresponding virtual (**B**, **D**, **F**) radiographs of the pelvis. **A**, **B** Measurement of the lateral center-edge (LCE) angle of the right hip and the acetabular index (AI) of the left hip. **C**, **D** Measurement of the femoral head extrusion index (EI). **E**, **F** Measurement of the retroversion index (RI), defined as the percentage of the retroverted portion of the acetabulum (a) relative to the total acetabular opening (a + b), and the presence of a positive posterior wall sign and ischial spine sign. AW, Anterior wall (red dotted line); IS Ischial spine; PW Posterior wall (blue dotted line)

Fifty CR of the pelvis in the patient cohort (100 hips) and their corresponding MRI-based VR were evaluated. In addition, the VR of 53 hips of 53 asymptomatic participants was used to analyze deformity prevalence. Only one hip per patient was randomly chosen in this analysis to avoid within-subject correlation. Radiographic analysis included nine standard radiographic parameters of acetabular coverage, including the lateral center-edge (LCE) angle, acetabular index (AI), EI, anterior and posterior coverage of the femoral head, and acetabular version (cross-over sign with retroversion index (RI), PW sign, and ischial spine (IS) sign) (Fig. 4 and Supplementary Fig. S2). Radiographic assessment was performed using the validated, commercially available “hip2norm” software (University of Berne) [9]. Measurements were performed semiautomatically: the reader manually identified the relevant anatomical landmarks, and hip2norm then computed the radiographic parameters. To assess the effect of contrast agent from MR arthrography as a potential source of error when assessing radiographic parameters, we divided the cohort of 100 hips into 50 hips undergoing unilateral MR arthrography and 50 contralateral hips without intra-articular contrast. Because the contrast and non-contrast groups corresponded to symptomatic and contralateral hips, respectively, this comparison reflects differences between symptomatic and contralateral hips rather than a controlled contrast experiment, as differences may therefore reflect laterality, disease severity or indication rather than intra-articular contrast alone.

All measurements were performed by an orthopedic surgery resident (JR) on both CR and VR in two independent reading sessions separated by more than 30 days. To assess interobserver reliability, a radiology resident (MM) with 5 years of experience independently evaluated the same set of CR and VR images, blinded to the initial analysis. The primary CR–VR comparison was based on the first reader’s (JR) measurements. The second reader (MM) provided only interobserver data, and intraobserver agreement was assessed for the first reader (JR). Both readers were blinded to clinical diagnosis and prior measurements, and CR and VR were assessed in separate sessions in random order. To evaluate whether radiographic diagnoses of acetabular deformities differ between CR and VR-based analysis, 100 hips were categorized into four diagnostic groups using previously validated thresholds: DDH (LCE < 23°), normal coverage (LCE 23 to 33°), acetabular overcoverage (LCE angle > 33°) and acetabular retroversion (combination of a cross over sign with RI > 30% and a positive PW sign) [10].

### Statistical analysis

Intra- and interobserver agreement for continuous parameters was assessed using the intraclass correlation coefficient (ICC) and 95% confidence intervals (CI), while binary parameters were evaluated using Cohen *κ*. Agreement thresholds were: ICC/*κ* < 0.20, slight; 0.21–0.40, fair; 0.41–0.60, moderate; 0.61–0.80, substantial; > 0.80, almost perfect [11]. Normality was assessed using the Kolmogorov–Smirnov test. Paired *t*-tests were applied, except for the non-normally distributed RI, which was analyzed with the Wilcoxon signed rank test. Bland–Altman plots assessed bias with 95% limits of agreement. Fixed bias was defined as the systematic mean difference between CR and VR (tested against zero using a one-sample *t*-test). The standard deviation of the interobserver difference on CR was used as a pragmatic endpoint to define the clinical relevance threshold for discrepancies between CR and VR (± 1 standard deviation), as variations that do not exceed interobserver bias may be clinically acceptable [6]. Changes in radiographic diagnosis were analyzed using the Stuart–Maxwell test, and overall agreement was quantified with Cohen *κ* using the interobserver agreement on CR as the reference. To address the within-subject correlation from including both hips, a sensitivity analysis was performed for the diagnostic classification by randomly selecting one hip per patient, repeated 1000 times, and reporting the median *κ* with 95% CI. Analyses were performed in R (version 4.3.2; R Foundation for Statistical Computing) with *α* = 0.05. The lateral center-edge angle was the primary outcome and was also used to stratify hips by acetabular coverage. The remaining radiographic parameters and version signs are declared secondary, exploratory endpoints. No adjustments for multiple comparisons were made. No formal sample-size or power calculation was performed.

## Results

### Patient and asymptomatic participants characteristics

From an initial sample of 115 patients, 65 patients were excluded due to previous hip surgery, as summarized in Fig. 1. The final study cohort included 50 patients (100 hips, 62% female) with a mean age of 34 ± 12 years (range, 18–58 years). Of these 50 patients, 21 (42%) underwent subsequent surgical treatment of the symptomatic hip, with arthroscopic FAI surgery being the most common procedure (15 patients, 30%). Fifty-three hips of 53 asymptomatic participants were included. Age was 29 ± 6 years (mean ± standard deviation; range, 19–43 years), and 28 participants (53%) were female.

### Interobserver and intraobserver agreements for CR in patients

Interobserver agreement for CR was almost perfect for five of the nine radiographic parameters, with ICC/κ values ranging from 0.82 (95% CI 0.70 to 0.93) for the IS sign to 0.93 (95% CI 0.89 to 0.96) for the LCE angle (all *p* < 0.001). The AI and PW sign showed substantial agreement, with ICCs of 0.76 (95% CI 0.60 to 0.85) and 0.68 (95% CI 0.54 to 0.82), respectively (Table 1). Intraobserver agreement for CR was almost perfect for all radiographic parameters, ranging from an ICC/κ of 0.83 (95% CI 0.70 to 0.96) for the cross-over sign to 0.96 (95% CI 0.93 to 0.97) for the LCE angle (all *p* < 0.001) (Table 1).

**Table 1:** Inter- and intraobserver agreement for standard radiographic parameters measured on conventional and virtual pelvic radiographs

Parameter	LCE	Acetabular index	Extrusion index	Retroversion index	Anterior coverage	Posterior coverage	Cross over sign	Posterior wall sign	Ischial spine sign
***Interobserver agreement: Intraclass correlation coefficient/Cohen’s κ***									
CR	0.93 (0.89–0.96)	0.76 (0.60–0.85)	0.86 (0.31–0.95)	0.87 (0.81–0.91)	0.83 (0.75–0.88)	0.82 (0.75–0.88)	0.84 (0.73–0.96)	0.68 (0.54–0.82)	0.82 (0.70–0.93)
Interobserver difference^a^	0.78 ± 2.13 (0.36–1.19)	1.48 ± 3.12 (0.86–2.09)	-2.24 ± 2.13 (-2.66 to -1.83)	0.31 ± 8.01 (-1.26 to 1.88)	-1.22 ± 4.32 (-2.06 to -0.37)	0.5 ± 4.8 (-0.44 to1.43)	-	-	-
VR with contrast	0.81 (0.65–0.90)	0.52 (0.19–0.73)	0.68 (0.13–0.86)	0.63 (0.40–0.78)	0.69 (0.51–0.81)	0.83 (0.71–0.90)	0.86 (0.68–0.86)	0.76 (0.58–0.94)	0.94 (0.81–1)
VR without contrast	0.73 (0.49–0.85)	0.31 (0.05–0.53)	0.60 (0.06–0.82)	0.81 (0.69–0.89)	0.88 (0.79–0.93)	0.82 (0.70–0.89)	0.75 (0.52–0.98)	0.86 (0.72–1)	0.73 (0.49–0.98)
***Intraobserver agreement: Intraclass correlation coefficient/Cohen’s κ***									
CR	0.96 (0.93–0.97)	0.90 (0.86–0.93)	0.91 (0.87–0.94)	0.96 (0.94–0.97)	0.86 (0.79–0.90)	0.87 (0.81–0.91)	0.83 (0.70–0.96)	0.92 (0.84–1.00)	0.84 (0.73–0.95)
VR with contrast	0.91 (0.85–0.95)	0.52 (0.28–0.70)	0.84 (0.73–0.91)	0.93 (0.88–0.96)	0.82 (0.71–0.90)	0.79 (0.66–0.88)	0.88 (0.71–1)	0.96 (0.88–1)	0.94 (0.81–1)
VR without contrast	0.92 (0.85–0.95)	0.40 (0.15–0.61)	0.88 (0.80–0.93)	0.91 (0.85–0.95)	0.89 (0.813–0.935)	0.887 (0.81–0.93)	0.86 (0.68–1)	0.95 (0.86–1)	0.93 (0.79–1)

Values for ICC or *κ* are given with 95% confidence intervals

*LCE* Lateral center edge, *CR* Conventional radiograph, *VR* Virtual radiograph

^a^ Differences for continuous parameters measured on conventional radiographs are reported as mean ± standard deviation and 95% confidence intervals

### Interobserver and intraobserver agreements for VR in patients

For most radiographic parameters on VR, interobserver agreement ranged from moderate to almost perfect (ICC/κ 0.60 to 0.94, all *p* < 0.001). The exception was the AI, which showed only fair agreement (ICC 0.52 [95% CI 0.19–0.73] with contrast; 0.31 [95% CI 0.05–0.53] without contrast). Intraobserver agreement was substantial to almost perfect for most parameters except the AI, which again showed only fair agreement (ICC 0.52 [95% CI 0.28–0.70] with contrast; 0.40 [95% CI 0.15–0.61] without contrast) (Table 1).

### Difference in radiographic parameters between CR and VR in patients

**Fig. 5:**
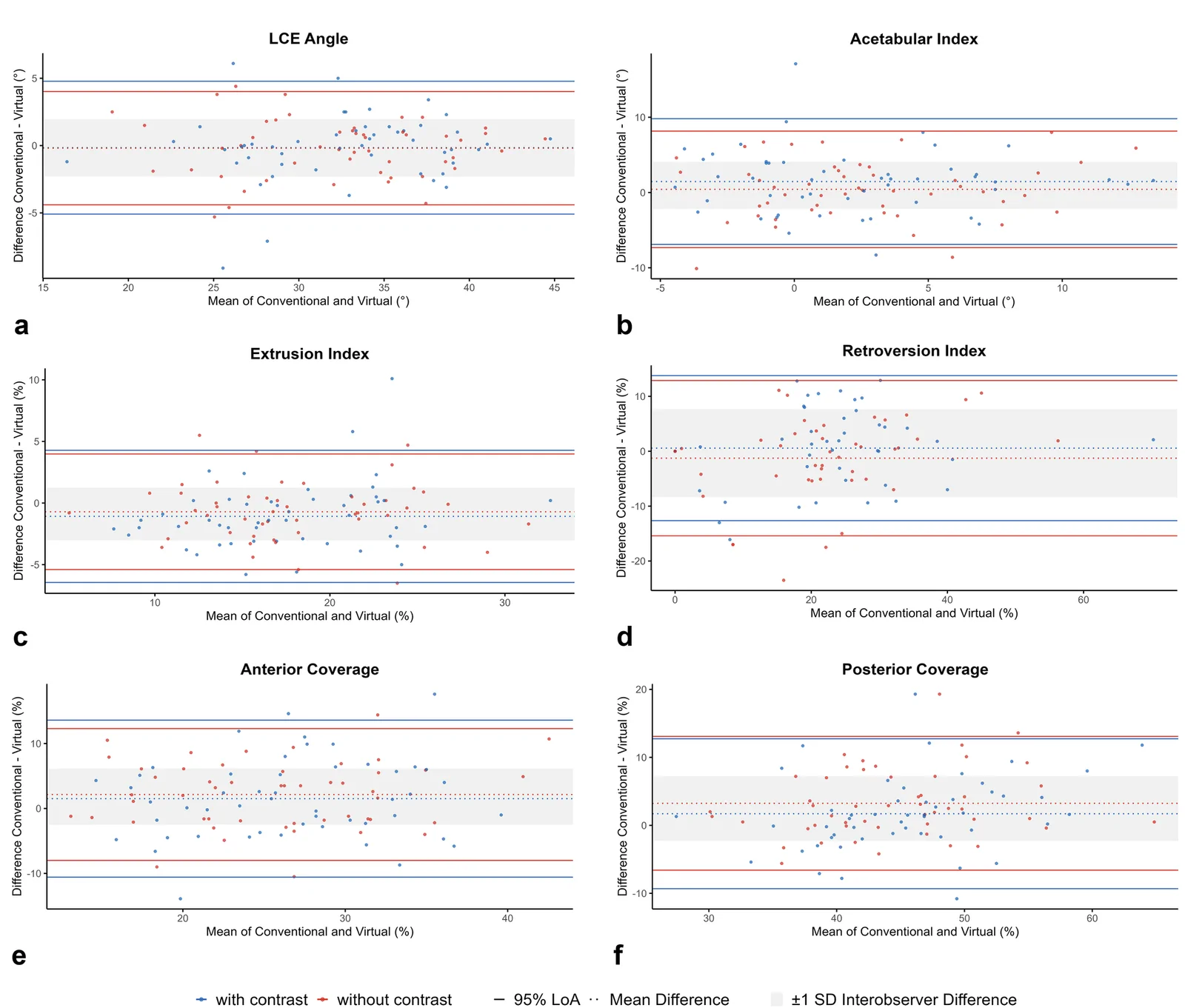
Bland–Altman analysis comparing conventional radiographs and VR for continuous parameters. **a** Lateral center edge angle, (**b**) acetabular index, (**c**) extrusion index, (**d**) retroversion index, (**e**) anterior coverage, (**f**) posterior coverage. Systematic bias was observed for VR with contrast (blue) for the acetabular index (mean difference 1.5%, *p* = 0.020), extrusion index (–1.1%, *p* = 0.007), and posterior coverage (1.7%, *p* = 0.037), and for VR without contrast (red) for the extrusion index (-0.7%, *p* = 0.042) and both femoral head coverage parameters: anterior (2.1%, *p* = 0.005) and posterior (3.2%, *p* < 0.001). Limits of agreement (LoA) were comparable between VR with/without contrast and CR; for example, for the LCE angle, LoA were -5.1 to 4.8° with contrast and -4.4 to 4.0° without contrast. The proportion of measurements within ±1 standard deviation of the interobserver difference (Table 2) on conventional radiographs (shaded area) was 72% (± 2.1°) for the (**a**) lateral center edge angle, 57% (± 3.1°) for the (**b**) acetabular index, 66% (± 2.1°) for the (**c**) extrusion index, 75% (± 8%) for the (**d**) retroversion index, 58% (± 4.3%) for (**e**) anterior coverage and 66% (± 4.3%) for (**f**) posterior coverage. LoA, Limits of agreement

Four out of nine radiographic parameters measured on VR with contrast showed fixed bias compared to the CR reference (Table 2 and Fig. 5). This included the AI (mean difference, 1.5 ± 4.3°, 95% CI 0.2 to 2.7°; *p* = 0.020), the extrusion index (EI) ( − 1.1 ± 2.7%, −1.9 to −0.3%; *p* = 0.007), IS sign (36% *versus* 18%, *p* = 0.043), and posterior coverage (1.7 ± 5.6%, 0.1 to 3.3%; *p* = 0.037). Four out of nine parameters differed as well between VR generated from hips without contrast and the CR reference (Table 3 and Fig. 5). This included the EI ( − 0.7 ± 2.4%, 95% CI, −1.4 to 0%; *p* = 0.042) and the IS sign (36% *versus* 14%; *p* = 0.011) and femoral head coverage parameters.

**Table 2:** Results of the comparison of standard radiographic parameters measured on conventional and MRI-based virtual pelvic radiographs for hips with intra-articular contrast (see also Fig. 5 for Bland–Altman analysis)

Parameter	Conventional radiograph	Virtual radiograph	Mean difference	*p*-Value	Limits of agreement
LCE [°]	32.5 ± 6	32.7 ± 5.6	-0.2 ± 2.5 (-0.9 to 0.6)	0.66	-5.1 to 4.8
Acetabular index [°]	2.9 ± 4.7	1.5 ± 4.9	1.5 ± 4.3 (0.2–2.7)	**0.02**	-6.9 to 9.8
Extrusion index [%]	17.0 ± 5.7	18.0 ± 5.1	-1.1 ± 2.7 (-1.9 to 0.3)	**0.01**	-6.4 to 4.3
Retroversion index [%]	20.2 ± 15.0	19.7 ± 13.9	0.6 ± 6.7 (-1.4 to 2.5)	0.50	-12.6 to 13.8
Anterior coverage [%]	27.4 ± 7.4	25.9 ± 6.7	1.5 ± 6.2 (-0.2 to 3.3)	0.09	-10.6 to 13.6
Posterior coverage [%]	46.4 ± 8.4	44.7 ± 6.9	1.7 ± 5.6 (0.1–3.3)	**0.04**	-9.3 to 12.8
Cross-over sign [%]	76	82	-	0.46	-
Posterior wall sign [%]	56	57	-	0.84	-
Ischial spine sign [%]	36	18	-	**0.04**	-

Continuous parameters are expressed as mean ± standard deviation and 95% confidence interval, **bold** indicates *p* < 0.05

*LCE* Lateral center egdge, *MRI* Magnetic resonance imaging

**Table 3:** Results of the comparison of standard radiographic parameters measured on conventional and MRI-based virtual pelvic radiographs for hips without contrast (see also Fig. 5 for Bland–Altman analysis)

Parameter	Conventional radiograph	Virtual radiograph	Mean difference	*p*-value	Limits of agreement
LCE [°]	32.2 ± 6.0	32.4 ± 6.0	-0.2 ± 2.2 (-0.8 to 0.4)	0.53	-4.4–4.0
Acetabular index [°]	2.8 ± 4.7	2.3 ± 4.4	0.4 ± 4.0 (-0.7 to 1.5)	0.45	-7.3 to 8.2
Extrusion index [%]	17.5 ± 5.6	18.2 ± 5.7	-0.7 ± 2.4 (-1.4 to 0)	**0.04**	-5.4 to 4.0
Retroversion index [%]	18.2 ± 14.5	19.4 ± 13.0	-1.3 ± 7.2 (-3.3 to 0.8)	0.49	-15.4 to 12.9
Anterior coverage [%]	26.6 ± 7.5	24.5 ± 7.1	2.1 ± 5.2 (0.7–3.6)	**0.01**	-8.0 to 12.3
Posterior coverage [%]	46.1 ± 7.9	42.9 ± 7.0	3.2 ± 5.0 (1.8–4.7)	**< 0.001**	-6.6 to 13.1
Cross-over sign [%]	76	82	-	0.46	-
Posterior wall sign [%]	64	68	-	0.67	-
Ischial spine sign [%]	36	14	-	**0.01**	-

Continuous parameters are expressed as mean ± standard deviation and 95% confidence interval,  **bold **indicates *p* < 0.05

*LCE* Lateral center edge, *MRI* Magnetic resonance imaging

Importantly, LCE angle measurements were comparable between VR with/without contrast and CR with mean differences of −0.2 ± 2.5° ( − 0.9 to 0.6°, *p* = 0.663) and −0.2 ± 2.2° ( − 0.8 to 0.4°, *p* = 0.530), respectively (Tables 2 and 3; Fig. 5). Furthermore Bland-Altman analysis revealed overlapping limits of agreement for quantitative parameters when measured on VR generated from hips with and without contrast. For example, limits of agreement were -5.1 to 4.8° (Table 2) and -4.4 to 4° (Table 3) for LCE angles derived from VR from hips with and without contrast, respectively. The comparable limits of agreement suggest that measurement variation was not clearly explained by intra-articular contrast. Because the groups comprised different hips, a contrast effect cannot be definitively excluded. Most differences between CR and VR measurements were within the range of ± 1 standard deviation of the interobserver variation of CR. This ranged from 57% for the AI ( ± 3.1° interobserver variation) to 75% for the RI ( ± 8% interobserver variation) (Fig. 5). Among the other continuous parameters, proportional bias was absent except for the RI (slope 0.10, *p* = 0.047) and posterior coverage (slope 0.18, *p* = 0.020).

### Changes in radiographic diagnosis of acetabular deformities in patients

On CR, the cohort comprised 8 dysplastic, 41 normally covered, 33 overcovered and 18 retroverted hips (Table 4). Diagnostic classification between CR and VR showed substantial agreement (*κ *= 0.62, 95% CI: 0.49 to 0.74), similar to the interobserver agreement of CR (*κ *= 0.70, 0.58 to 0.81) as indicated by the overlapping CIs. Furthermore, there was no difference in stratification of hip deformities (*p* = 0.918) (Table 4). Of 100 hips, 26 were classified differently on VR than CR, in both directions and mostly in hips close to the diagnostic thresholds.

**Table 4:** Radiographic diagnosis of hip morphology using conventional and virtual pelvic radiographs in the patient analysis and virtual radiographs in asymptomatic participants

Diagnosis	CR	VR	CR Reader 1	CR Reader 2	VR
Cohort	**Patients**	**Patients**	**Patients**	**Patients**	**Asymptomatic participants**
Dysplasia (hips)	8	8	8	11	7
Normal (hips)	41	44	41	44	36
Overcoverage (hips)	33	31	33	27	10
Retroversion (hips)	18	17	18	18	0
Total (hips)	100	100	100	100	53
Overall *p*-value	0.92		0.10		n.a.
Overall agreement (*κ*)	0.62 (0.49–0.74) *p* < 0.001		0.70 (0.58–0.81) *p *< 0.001		n.a.

*κ*-values are given with 95% confidence intervals

*CR* Conventional radiograph, *VR* Virtual radiograph

### Radiographic diagnosis of acetabular deformities in asymptomatic participants

**Fig. 6:**
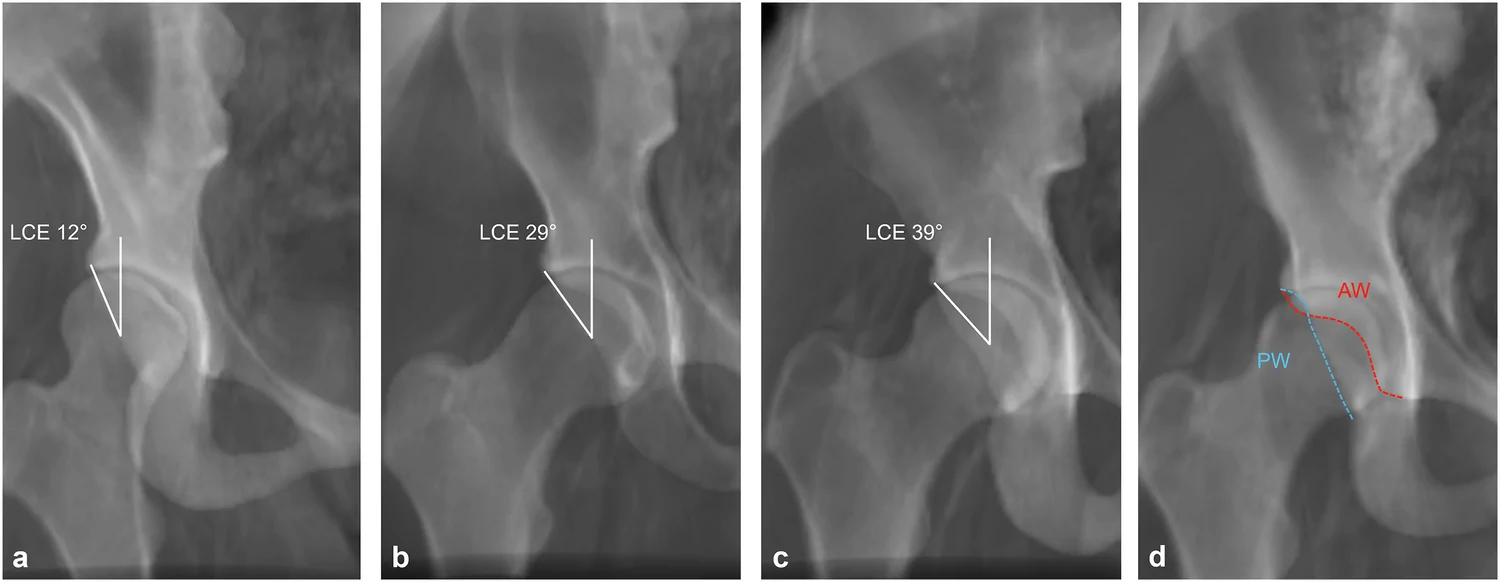
Virtual pelvic radiographs of asymptomatic participants (right hip shown). Radiographic assessment demonstrated normal acetabular coverage in the majority of hips (**b**; 36 hips, 68%), while 7 hips (13%) showed acetabular dysplasia (**a**) and 10 hips (19%) showed acetabular overcoverage (**c**). Although apical acetabular retroversion was observed (**d**), no hip met the diagnostic criteria for retroversion (positive cross-over sign with a retroversion index > 30% in combination with a positive posterior wall sign). LCE, Lateral center edge angle; AW, Anterior wall (red dotted line); PW, Posterior wall (blue dotted line)

Among the 53 asymptomatic participants, the mean LCE angle as assessed on VR was 29 ± 5°, while the AI was 7 ± 3°. Overall, the majority showed normal acetabular coverage (36 hips, 68%) while 7 hips (13%) showed acetabular dysplasia and 10 hips (19%) had acetabular overcoverage (Fig. 6 and Table 4).

## Discussion

This study demonstrates that MRI-based VR of the pelvis, generated through a deep-learning segmentation pipeline combined with the generation of pseudo-CT images and cone-beam projection, constitutes a feasible and accurate adjunct for assessing acetabular morphology.

The reliability assessment revealed that VR measurements demonstrated robust intra- and interobserver agreement across most parameters, with ICC/k values ranging from 0.60 to 0.96, indicating moderate to almost perfect reliability. However, the AI consistently showed the poorest agreement (ICC 0.52 and 0.31 for hips with contrast, and 0.52 and 0.40 for hips without contrast), suggesting that this parameter is particularly vulnerable to the technical limitations of the VR approach. However, the interobserver agreement for AI (ICC of 0.76) was also lower compared to other parameters when evaluated on the reference CR (Table 1). This likely reflects its dependence on precise identification of the medial sclerotic line, which may be further complicated by VR. This is a subtle anatomical landmark compared to other parameters, such as the LCE angle, which relies on the more prominent appearance of the lateral sclerotic acetabular rim and yielded higher interobserver agreement (ICC ranging from 0.73 to 0.92 for VR). Comparison between CR and VR revealed significant differences in four out of nine parameters of acetabular coverage and version for VR. This measurement bias likely stems from several factors. As demonstrated in Fig. 4, VRs exhibit less sharp edges and somewhat blurred contours. This is related to the considerably lower in-plane resolution of our MRI input sequence (1.2 mm²) compared to CR (0.2–0.3 mm²) [12]. Furthermore, our approach for the generation of pseudo-CT images cannot fully replicate the attenuation-based contrast of CR. These factors likely affect parameters requiring precise contour definition, such as the AI, for which we observed a mean difference of 1.5 ± 4.3° generated from VR with contrast. Importantly, however, the majority of the differences observed between both modalities fell within the range of interobserver variation, suggesting that these discrepancies are within a clinically acceptable range. Although several parameters showed statistically significant bias, the magnitudes were small relative to diagnostic threshold ranges and to CR interobserver variation and are unlikely to alter diagnostic classification except in hips close to thresholds. The differing prevalence of the positive IS sign between CR and VR likely reflects differences in projection geometry and ischial-spine depiction, consistent with the known sensitivity of version signs to pelvic tilt and rotation [13].

Zero echo time (ZTE) or ultrashort echo time (UTE) MRI could improve cortical bone visibility for VR generation, as recent work demonstrates CT-like bone-to-background contrast from ZTE pelvis sequences [14]. However, acquisition times compatible with routine clinical use remain unavailable, limiting current feasibility

The LCE angle, recognized as the most clinically significant parameter for evaluating acetabular coverage [5, 6], demonstrated no statistically significant differences between VR and CR. Accordingly, substantial agreement (*κ* = 0.62) in the stratification of acetabular deformities between CR and VR was achieved. This was comparable to substantial agreement between the two readers when evaluating acetabular deformities on CR (*κ *= 0.70). This consistency is critically important, as diagnosis of acetabular deformities using radiographic parameters serves as the foundation for diagnostic evaluation in joint-preserving hip surgery [10, 15]. We further acknowledge that the LCE angle alone does not fully capture the complexity of acetabular morphology and that alternative classification systems and thresholds, including those defining borderline dysplasia, have been proposed [16, 17]. Therefore, hips with LCE values close to the 23° cut-off should be interpreted with caution. We acknowledge that MRI is a more resource-intensive modality than standard radiography, and its availability and cost vary substantially across healthcare systems. However, the value lies not in replacing the initial diagnostic radiograph, but in the “one-stop-shop“ capability of the MRI protocol. Since patients with symptomatic hip deformities routinely undergo MRI for soft tissue assessment and 3D surgical planning, generating a VR from existing data adds value without additional cost or radiation. The proposed workflow is not intended to mandate additional MRI but to extract radiographic parameters from MRI already acquired for soft-tissue assessment and 3D planning. In settings where MRI is not routinely available, CR remains the appropriate first-line modality. Our results support the potential adoption of VRs in clinical scenarios in which radiation exposure presents significant concerns, including young patients requiring serial imaging or population-based screening programs.

The application of VR in our cohort of 53 asymptomatic participants demonstrated the clinical applicability of this radiation-free approach to assessing acetabular morphology, despite being exploratory due to the lack of CR reference. The observed prevalence of acetabular dysplasia (13% using LCE < 23°) falls within the range reported in population-based studies using CR, where prevalence varied substantially depending on the threshold applied in a cohort of 2072 young adults. This ranged from3.3% (LCE angle < 20°) to 20% (LCE < 25°) [18]. Generating VR from clinical MRI protocols expands the potential utility of this technique beyond symptomatic patient populations to research settings investigating the epidemiology and natural history of hip deformities. Furthermore, the absence of ionizing radiation makes this approach suitable for longitudinal monitoring and serial imaging in young patients, where cumulative radiation dose remains a concern. Studies with larger asymptomatic cohorts could establish normative values for VR-based acetabular parameters across age groups and populations.

The retrospective initial feasibility study has several limitations. First, VRs were generated from a single sequence with lower in-plane resolution compared to CR. However, the 3D T1-weighted VIBE Dixon sequence is fast and enables accurate 3D pelvic bone segmentation and is part of our routine protocol. By contrast, high-resolution imaging with longer acquisition times was limited to the affected hip joint, as is commonly recommended [15, 19]. Future studies should evaluate whether VR assessment can be improved by using high-resolution pelvic images and enhanced bone contrast with ZTE or UTE MRI. Second, we excluded advanced osteoarthritis and complex deformities to first validate the technique on standard morphology. These complex pathologies present distinct segmentation challenges (*e.g*., bone loss, open physes) that require separate technical optimization before the VR technique can be generalized. Third, our findings may not apply to standing radiographs, as no consensus exists on their use for acetabular assessment, and many centers, including our institution, perform routine supine pelvic radiographs. Fourth, this was a single-center study performed in a selected tertiary hip-preservation center, which limits generalizability, and measurement agreement may differ in broader populations. Fifth, patient and asymptomatic cohorts were imaged on different 3 T systems (MAGNETOM Vida and Prisma). Although identical VIBE Dixon parameters were used, minor scanner-dependent effects cannot be entirely excluded. Sixth, acetabular retroversion was defined using a conservative triad that favored specificity over sensitivity, so some retroversion abnormalities may not have been captured [20]. As acetabular version signs are sensitive to pelvic tilt and rotation, residual differences in pelvic orientation between CR and VR cannot be excluded. Finally, the asymptomatic cohort was younger than the patient cohort. As the two groups were not directly compared, and acetabular morphology is largely stable in skeletally mature adults, this is unlikely to bias the descriptive prevalence estimates, although residual age effects cannot be excluded [21].

## Conclusion

In conclusion, this study demonstrates that virtual anteroposterior pelvic radiographs generated from clinical hip MRI using a deep learning-based approach represent a feasible and clinically comparable adjunct for assessing acetabular morphology, albeit with limitations due to the poor reliability and reproducibility of the AI. The successful application in asymptomatic participants, identifying clinically relevant morphology without radiation exposure, highlights the potential of this technique for population-based research where CR is precluded. By enabling the extraction of standard 2D metrics from 3D MRI datasets, this workflow offers a radiation-free, cost-effective enhancement to the assessment of hip deformities in both clinical and research settings.

## Supplementary information

**Additional file 1: Figure S1.** The 3D bone models of the pelvis are used as input for a cone-beam projection algorithm in which a virtual cone beam source and virtual detector plate are used to mimic the radiographic distortion of conventional radiographs. This cone beam projection leads to the more lateral projection of more anteriorly located structures such as the anterior acetabular wall (blue) compared to more posteriorly located structures (red). **Supplementary Figure S2.** Nine radiographic parameters assessed on conventional and VR: (A) LCE angle (formed by line through center and most lateral acetabular edge and a vertical line), (B) acetabular index (formed by line through medial and lateral edge of weight bearing zone and a horizontal line), (C) extrusion index (percentage of uncovered portion of femoral head ‘a’ in relation to femoral diameter ‘a+b’), (D) retroversion index (percentage of retroverted acetabulum ‘a’ to acetabular opening ‘a+b’), (E) cross over sign (positive when anterior wall [AW] crosses posterior wall [PW]), (F) posterior wall sign (positive when posterior wall medial to femoral head center), (G) ischial spine sign (positive if ischial spine medial to iliopectineal line), (H) anterior coverage (percentage of femoral head covered by the anterior acetabular rim) and (I) posterior coverage (percentage of femoral head covered by the posterior acetabular rim.

## Data Availability

The segmentation algorithms and datasets used and/or analyzed during the current study are available from the corresponding author upon reasonable request.

## Abbreviations

2D: Two-dimensional
3D: Three-dimensional
AI: Acetabular index
AW: Anterior wall
CR: Conventional radiographs
CI: Confidence interval
CT: Computed tomography
DDH: Developmental dysplasia of the hip
EI: Extrusion index
FAI: Femoroacetabular Impingement
IS: Ischial spine
LCE: Lateral center edge
PW: Posterior wall
RI: Retroversion Index
UTE: Ultrashort echo time
VIBE: Volume-interpolated breath-hold examination
VR: Virtual radiographs
ZTE: Zero echo time
