# Supplementary material for: MRI-based virtual radiographs of the pelvis for assessment of acetabular morphology
Source: Eur Radiol Exp. 2026 Nov 6;10:173. doi: 10.1186/s41747-026-00835-7 (PMC13743690; doi:10.1186/s41747-026-00835-7)

MRI- Based Virtual Radiographs of the Pelvis for Assessment of Acetabular Morphology

Electronic Supplementary Material

Supplementary Figure S1. The 3D bone models of the pelvis are used as input for a cone-beam projection algorithm in which a virtual cone beam source and virtual detector plate are used to mimic the radiographic distortion of conventional radiographs. This cone beam projection leads to the more lateral projection of more anteriorly located structures such as the anterior acetabular wall (blue) compared to more posteriorly located structures (red)

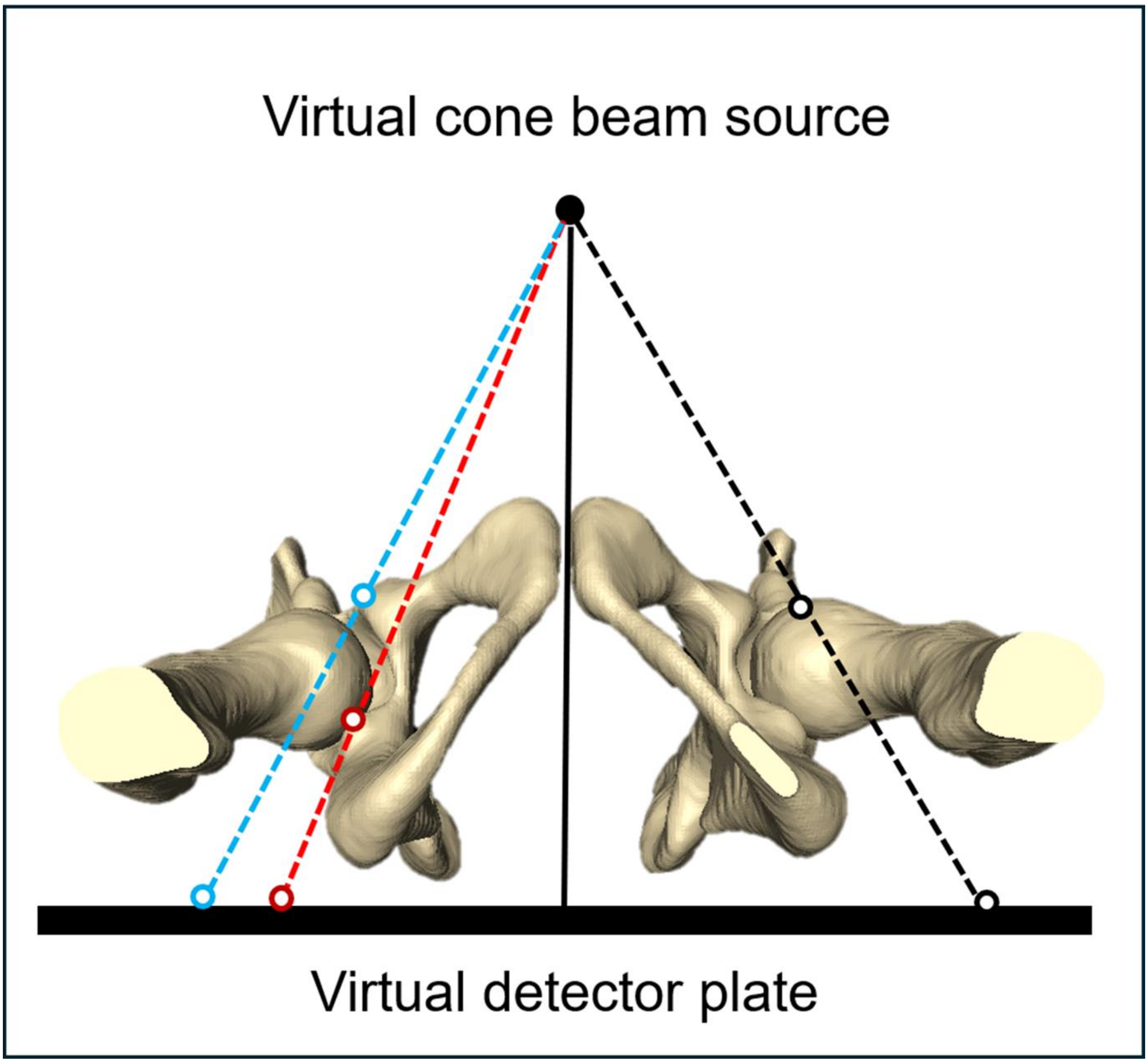

Supplementary Figure S2. Nine radiographic parameters assessed on conventional and VR: (A) LCE angle (formed by line through center and most lateral acetabular edge and a vertical line), (B) acetabular index (formed by line through medial and lateral edge of weight bearing zone and a horizontal line), (C) extrusion index (percentage of uncovered portion of femoral head 'a' in relation to femoral diameter 'a+b'), (D) retroversion index (percentage of retroverted acetabulum 'a' to acetabular opening 'a+b'), (E) cross over sign (positive when anterior wall [AW] crosses posterior wall [PW]), (F) posterior wall sign (positive when posterior wall medial to femoral head center), (G) ischial spine sign (positive if ischial spine medial to iliopectineal line), (H) anterior coverage (percentage of femoral head covered by the anterior acetabular rim) and (I) posterior coverage (percentage of femoral head covered by the posterior acetabular rim

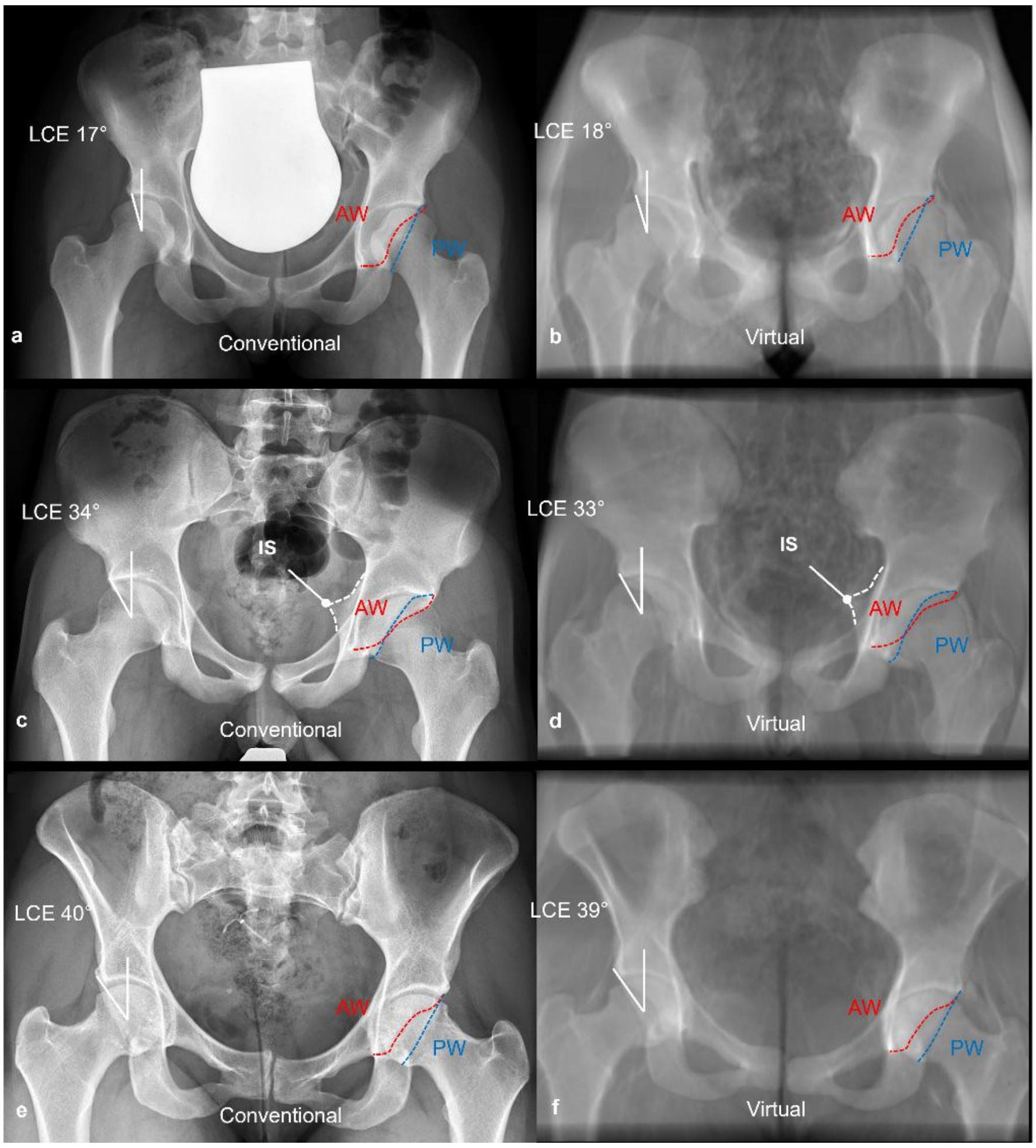

Supplement: Supplementary file 1 [file 41747_2026_835_MOESM1_ESM.pdf]
